# Association Between Physical Activity and Lower Risk of Lung Cancer: A Meta-Analysis of Cohort Studies

**DOI:** 10.3389/fonc.2019.00005

**Published:** 2019-01-22

**Authors:** Yang Liu, Yue Li, Yun-Peng Bai, Xiao-Xi Fan

**Affiliations:** Department of Thoracic Surgery, The First Affiliated Hospital of China Medical University, Shenyang, China

**Keywords:** physical activity, lung cancer, smoking, cohort study, meta-analysis

## Abstract

**Background:** Epidemiological evidences regarding the association between physical activity and the risk of lung cancer are still controversial.

**Objectives:** We aimed to investigate the relationship between physical activity and risk of lung cancer in men and women, as well as other high-risk populations such as cigarette smokers.

**Methods:** We conducted a meta-analysis of cohort studies to evaluate the association between physical activity and risk of lung cancer. Relevant studies were identified by searching PubMed and Web of Knowledge through August 2018. Study-specific relative risk (RR) with 95% confidence interval (CI) were pooled using random effect model when significant heterogeneity was detected.

**Results:**Twenty cohort studies with a total of 2,965,811 participants and 31,807 lung cancer cases were included. There was an inverse association between the physical activity and risk of lung cancer. Compared with the low level of physical activity, the pooled RR was 0.83 (95%CI: 0.77, 0.90), with significant heterogeneity (*I*^2^ = 62.6%, *P*_heterogeneity_ < 0.001). The corresponding pooled RRs were 0.90 (95%CI: 0.82, 0.99) for women and 0.81 (95%CI: 0.73, 0.90) for men. Smokers with a high level of physical activity were associated with a 10% lower risk for lung cancer (RR = 0.90, 95% CI: 0.84, 0.97), while the association was not significant among non-smokers (RR = 0.95, 95% CI: 0.88, 1.03). Subgroups analysis stratified by whether the studies adjusted for smoking intensity and durations yielded the same magnitude of RR. However, the RR for subgroups without adjustment for dietary factors was 0.74 (95%CI: 0.71, 0.77), which was significantly lower than that with dietary factors adjusted (RR = 0.89, 95%CI: 0.84, 0.95).

**Conclusions:**Increased physical activity might be associated with lower risk of lung cancer. Such inverse association was identified among smokers rather than non-smokers. Large interventional studies are expected to further verify these findings.

## Introduction

The global burden of lung cancer has been increasing over the past years. It is the most frequently diagnosed cancer and the leading cause of cancer death, accounting for 11.6% (2,093,876 new cases) of the total new cancer cases and 18.4% (1,761,007 deaths) of the total cancer deaths in 2018 (1). As such, primary prevention of lung cancer is therefore a critical public health challenge worldwide.

Cumulative observational evidences suggested that physical activity may be significantly associated with a reduced risk of lung cancer (2–7), while others did not observed such an association (8–12). Of note, it has been reported that physical activity was unrelated to lung cancer among never smokers but it was inversely associated with lung cancer among former and current smokers (13, 14). Previous meta-analysis with both cohort studies and case-control studies have detected an inverse association between physical activity and risk of lung cancer (14, 15). Since potential selection and recall bias related to the design of case-control study might distort the true association, the relationship is still unclear. Thus, the International Agency of Research in Cancer concluded that the association between physical activity and risk of lung cancer remained inconclusive (16). In addition, a protective effect of physical activity on lung cancer was categorized as limited evidence in the World Cancer Research Fund/American Institute for Cancer Research report from 2007–2018.

Recently, several large prospective cohorts have evaluated the association of physical activity with lung cancer (6, 7, 12, 17, 18). A prospective study of seven Australian cohorts with 3,67,058 participants reported a significantly inverse association in men, but not in women, suggesting a possible gender disparity for relationship (7). Moreover, the Physical Activity Collaboration of the National Cancer Institute's Cohort Consortium with 1.44 million adults reported that smoking status modified the association for lung cancer (6). It is still unclear whether the association between physical activity and lung cancer is the result of an underestimation of lifetime smoking; and therefore a better understanding of this association in never smokers is needed.

Thus, an integration with these most up-to-date evidences from these large cohorts may address these issues and make it possible to detect the association, as well as the possible effect modifications by smoking status and gender. Since evidences from large prospective cohort studies with less potential bias are more convince, we aimed at conducting an updated meta-analysis including only cohort studies to quantitatively assess the association between physical activity with risk of lung cancer.

## Materials and Methods

### Data Sources and Search Strategy

This meta-analysis was reported using the Preferred Reporting Items for Systematic reviews and Meta-Analyses guidelines (19). A literature search through August 2018 was performed using PubMed and Web of Knowledge with the combination of the following key words: (“physical activity” or “exercise”) and (“cancer” or “neoplasm” or “carcinoma” or “tumor”) and “lung” and “cohort.” In addition, we also manually searched the reference lists of relevant publications to identify additional studies.

### Studies Selections and Data Extraction

The study selection process consisted of title/abstract screening and full-text review. In the title/abstract screening stage, records were identified based on their relevance to the study topic, i.e., whether the study used a cohort design and presented the information on physical activity as the exposure of interest and incident lung cancer as the outcome of interest. In the full-text review stage, studies were included if they further provided relative risk (RR), hazard ratio or risk ratio of highest level vs. lowest level as risk estimates with the corresponding 95% confidence intervals (CIs) or standard errors. We used the RR as the measure of the association between physical activity and risk of lung cancer. If multiple estimates were provided, priority was given to the multivariable adjusted risk estimates. Instances in which data were insufficient or missing, we attempted to contact the authors of the articles to request the relevant data. Two authors (Yang Liu and Yue Li) independently performed the literature search and study selection. Discrepancies were resolved by discussion with other reviewer (Xiao-Xi Fan).

Two researchers (Xiao-Xi Fan and Yun-Peng Bai) independently used a standardized reporting form to abstract the following data from each study: the first author's name, the year of publication, the country in which the study was performed, the duration of follow-up, number of study population, the number of lung cancer events, the assessment of physical activity, the study outcome, the categories of exposure with the corresponding RRs and 95% CIs, the covariates adjusted in multivariable models.

### Quality Assessment

To assess study quality, a 9-point system on the basis of the Newcastle-Ottawa Scale was used in which a study was judged on 3 broad categories for cohort studies as follows: the selection of study groups, comparability of groups, and ascertainment of either the exposure or outcome of interest (20).

### Data Analysis

To examine associations between of physical activity and risk of lung cancer, we used the random effect model proposed by DerSimonian and Laird to pool the study specific estimates if significant heterogeneity was observed (21). Subgroup analyses were conducted to explore the potential sources of heterogeneity. Subgroup analyses were conducted stratified by gender, study location, size of cohort, exposure assessment, outcome assessment, smoking status, and whether the studies adjusted for potential confounders or risk factors. We also performed a sensitivity analysis of the influence of individual studies on the pooled estimates by repeating the meta-analysis excluding one study at a time. Heterogeneity among studies was assessed with the Q and the *I*^2^ statistics and results were defined as heterogeneous for a *P*-value < 0.10 or an *I*^2^ > 50% (22). Publication bias were evaluated by Begg's (23), Egger's tests (24) and arcsine-Thompson test (25). The trim and fill method was employed to adjust for potential publication bias (26).

Statistical analyses were conducted using Stata (version 13.0). Two-sided *P*-values of < 0.05 were considered statistically significant.

## Results

### Literature Search, Study Characteristics and Quality Assessment

**Figure 1:**
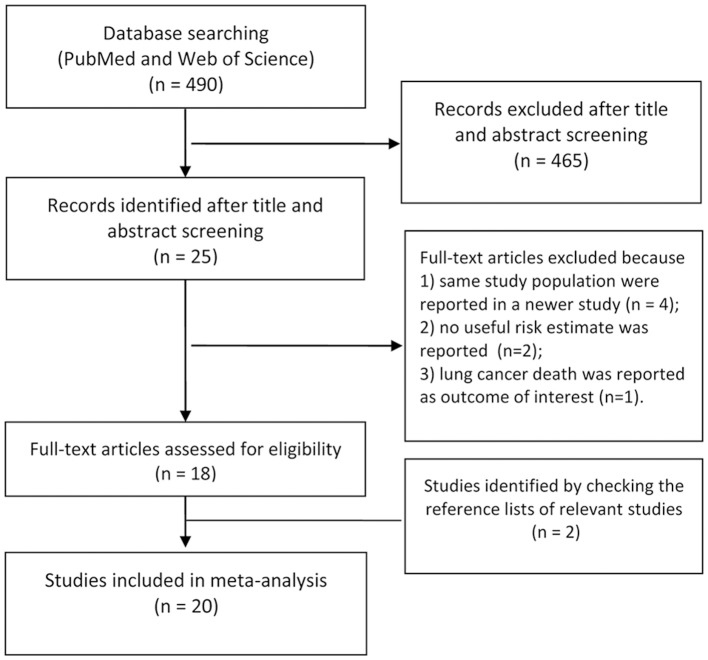
Flow chart of the selection of studies in this meta-analysis.

A flow diagram for the search is presented in Figure 1. A total of 490 records were identified from the 2 databases. After review of the titles and abstracts, 465 records were excluded because of little relevance to the study topic. After reviewing the full text of the remaining 25 cohort studies, four studies (27–30) were excluded as same study population were reported in the newer study; two studies (31, 32) were excluded as no RRs or 95% CIs were reported; One (33) was excluded because of reporting the lung cancer death as the outcome of interest. Two additional studies (5, 34) were identified by checking the reference lists of relevant articles. Thus, we included 20 cohort studies in the final analysis (2–12, 17, 18, 34–40).

Descriptive data for the 20 included cohort studies were summarized in Table 1. There was a total of 2,965,811 cohort members, of whom 31,807 developed lung cancer during follow-up. Eight studies were conducted in the United States, 8 studies in European countries, 2 in Asian countries, and 2 in others areas. The study population were adults with age of >18 years. Most studies were adjusted for major confounders including age, sex, smoking status, and body mass index, etc.

**Table 1:** Characteristics of studies included in the meta-analysis.

**References**	**Age (years)**	**Location**	**Study cohort**	**Size of cohort**	**Number of cases**	**Exposure measurement**	**Outcome measurement**	**Follow-up**	**Confounding adjustments**
Albanes et al. (35)	25–74	US	US National Health and Nutrition Examination Survey (NHANES I) and NHANES I Epidemiological Follow-up	5,138 men and 7,407 women	114	Non-recreational activity and recreational activity	hospital record or death certificate review	median: 10 years	Age, cigarette smoking status and pack-year history, economic status, reproductive and family breast cancer history, BMI, or dietary fat or energy intake
Severson et al. (36)	30+	US	Japan-Hawaii Cancer Study	8,006	194	Non-recreational activity and recreational activity	Cancer registry	1965–1986	Age, body mass index, cigarette smoking
Knekt et al. (34)	30+	Europe	Mini-Finland Health Survey	3,245	70	Leisure-time exercise	Cancer Registry	14 years	Age
Thune and Lund, (37)	20-49	Europe	Population based Health Survey	1,04,485 men and women	464	Leisure activity and work activity	Cancer Registry	1972–1991	Age at entry, geographical region, smoking habits, number of cigarettes smoked, years smoked and body mass index.
Lee et al. (2)	Mean: 58 years	US	The Harvard Alumni Health Study	13,905	245	Non-recreational activity and recreational activity	Self-reported and death certificates	1985–1997	Age, cigarette smoking, body mass index
Wannamethee et al. (38)	40–59	Europe	The British Regional Heart Study (BRHS)	7,588	265	Non-recreational activity and recreational activity	death certificates; cancer registry, postal questionnaires	Mean: 18.8 years	Age, smoking, body mass index, alcohol intake and social class
Colbert et al. (39)	50–69	Europe	Alpha-Tocopherol, Beta Carotene Cancer Prevention (ATBC) Study	29,133	1,442	Usual occupational and leisure time physical activity	Cancer Registry	1985–1997	randomization
Alfano et al. (10)	Mean: 63years	US	Beta-Carotene and Retinol Efficacy Trial (CARET)	7,405	263	Sleeping, vigorous activity, moderate activity, light activity, and sitting	cancer registries, state boards of health, and the National Death Index	<5 years	Age, education level, ethnicity, gender, marital status, employment, and household structure, Health-Related Variables
Schnohr et al. (9)	20–93	Europe	The Copenhagen Center for Prospective Population Studies	28,000	228	Leisure-time physical activity	Cancer registry and National Central Person Register	14 years	Age, birth cohort, cohort membership and occupational physical activity, smoking, education and alcohol consumption, duration of smoking, and interaction between smoking status and duration
Sprague et al. (3)	43–86	US	University of Wisconsin Extension-Survey Research Laboratory	4,831	134	Total physical activity	Cancer Registry, death certificates, and the National Death Index	12.8 years	Age, sex, pack-years of smoking, time since smoking cessation, body mass index, alcohol intake, and education
Yun et al. (4)	40+	Asia	The National Health Insurance Corporation Study (NHICS)	4,44,963	1,574	Leisure-time physical activity	Cancer registry	6 years	Age, dietary preference, LPA, smoking status, amount of alcohol drinking, body mass index, employment and fasting blood sugar as appropriate
Inoue et al. (8)	45–74	Asia	Japan Public Health Center-based Prospective Study	79,771	532	Total physical activity	notification from the major hospitals in the study area, cancer registries, and Death certificates	1995–2004; Average: 7.5 years	Age, area, total energy intake, history of diabetes, smoking status, alcohol intake status, body mass index, and leisure-time sports or physical exercise.
Laukkanen et al. (5)	42–60	Europe	Kuopio Ischemic Heart Disease Risk Factor Study	2,268	52	Total physical activity	Cancer registry and death registry	16.7 years	Age and examination year, cigarette smoking, alcohol consumption, waist-to-hip ratio, SES and total caloric, fiber and fat intake.
Land et al. (11)	<65 years (>80% subjects)	US	The National Surgical Adjuvant Breast and Bowel Project (NSABP) Breast Cancer Prevention Trial (P-1) NSABP-1	13,388	66	Leisure-time physical activity	clinical examinations and pathology reports	7 years	Randomization
Sormunen et al. (40)	35–94	Europe	Finland Athletic Sample	2,448	87	Leisure-time physical activity	Cancer registry	1986–2010	Age, smoking status and pack-years of smoking
Moore et al. (6)	19–98	US and Europe	The Physical Activity Collaboration of the National Cancer Institute's Cohort Consortium	1,436,624	19,133	Leisure-time physical activity	Multiple methods	median: 11 years	Age, sex, smoking status, alcohol consumption, education, and race/ethnicity.
Wang et al. (17)	50–79	US	Women's Health Initiative Observational Study (WHI-OS) and Clinical Trial (WHI-CT)	1,29,401	2,148	Leisure-time physical activity	medical and pathology records review	11.8 years	Age, race/ethnicity, BMI, family history of cancer, personal history of cancer, history of asthma, history of emphysema or chronic bronchitis, smoking, education, alcohol intake, vitamin D use, hormone therapy, oral contraceptive use, hysterectomy status, NSAID use, servings of fruit, vegetables, and red meat
Patel et al. (18)	50–74	US	CPS-II Nutrition Cohort	1,84,185	1,905	Leisure-time physical activity	medical records and linkage with state cancer registries	14 years	Age, sex, race, sitting time, marital status, prevalent disease (emphysema or other lung diseases), education, cigarettes per day and smoking duration, fruits and vegetables consumption, and Body mass index
Laaksonen et al. (7)	18+	Australia	Australian cancer-PAF cohort consortium	3,67,058	2,025	Leisure-time physical activity	Australian Cancer Database and National Death Index	10 years	Age, sex, study, smoking, fruit consumption
Borch et al. (12)	30–70	Europe	Norwegian Women and Cancer Study (NOWAC)	86,499	866	Total physical activity	Cancer Registry	12.9 years	Body mass index, years of education, smoking status and pack-years, fruit consumption, birth cohort

The quality scores ranged from 5 to 9 with a median score of 8. Three studies were evaluated with a score of <7, and others with a score of ≥7. Thus, the majority of the studies included in the meta-analysis were assessed as high-quality studies (Supplementary Table 1).

### Synthesized Findings

**Figure 2:**
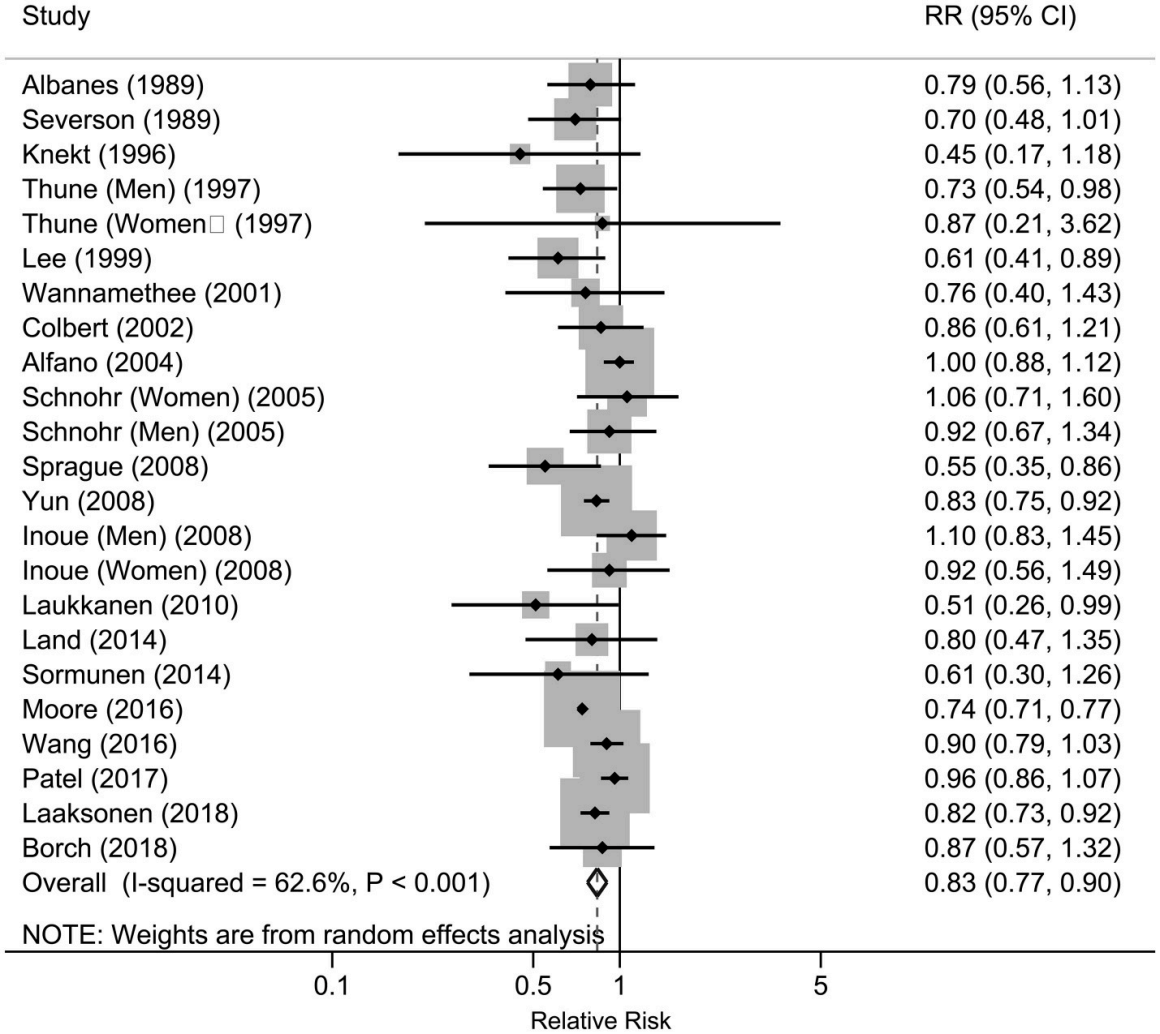
Forest plot of the associations between physical activity and risk of lung cancer, 1989–2018.

**Figure 3:**
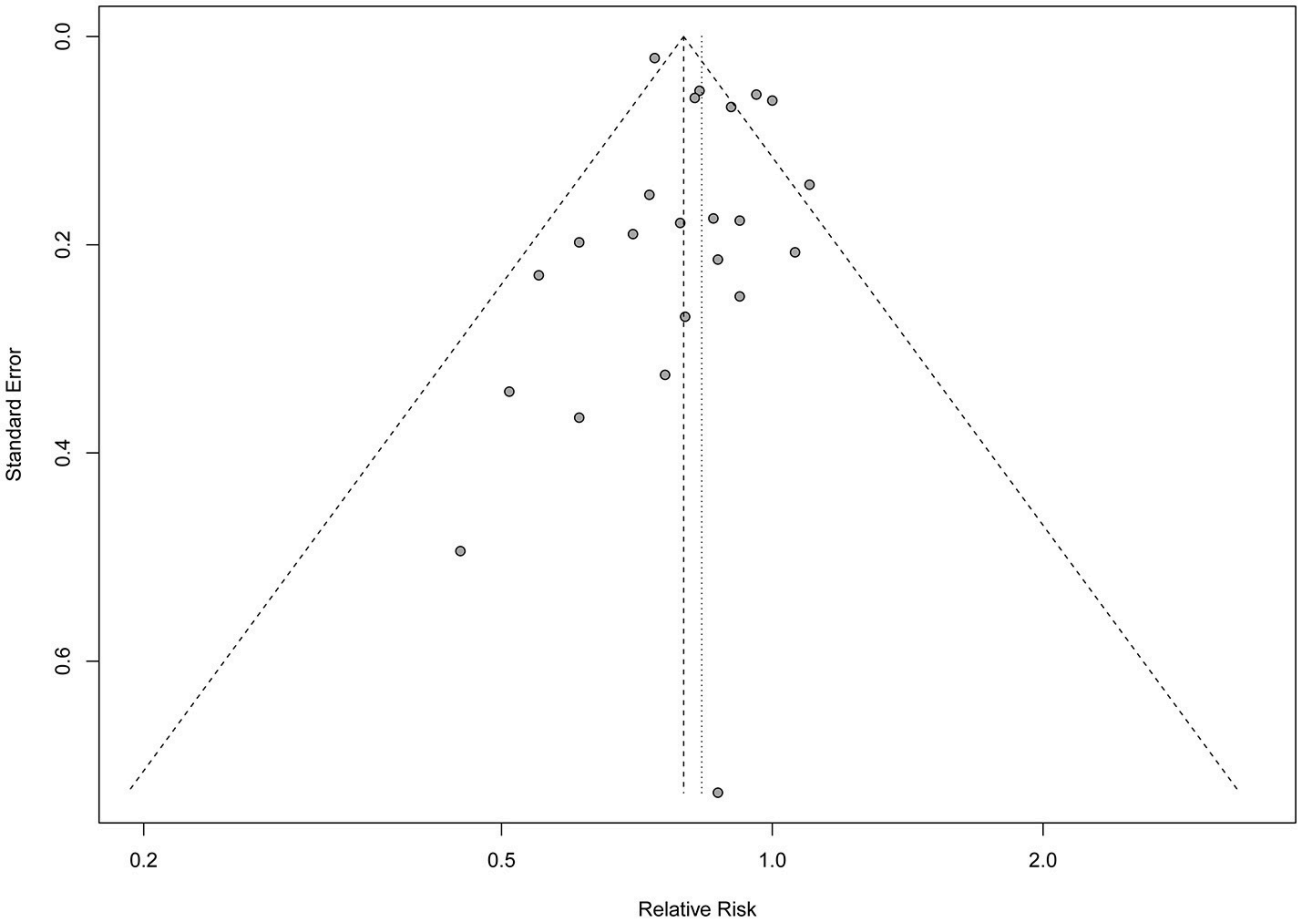
Funnel plot of the meta-analysis.

As shown in Figure 2, seven studies reported statistically significant inverse associations and the others showed null associations. The overall analysis of the 20 studies showed an inverse association between the physical activity and risk of lung cancer, which indicated that increased physical activity may be associated with a lower the risk of lung cancer. Compared with the low level of physical activity, the pooled RRs were 0.83 (95%CI: 0.77, 0.90), with significant heterogeneity (*I*^2^ = 62.6%, *P*_heterogeneity_ < 0.001) across the included studies. Publication bias were tested by Egger's test (*P* = 0.414), Begg's test (*P* = 0.023) and arcsine-Thompson test (*P* = 0.151). The funnel plot was shown in Figure 3. Furthermore, when attempting to use the trim and fill method to adjust for potential publication bias, no additional studies have been added, resulting in it having the same pooled RR and 95% CI as the original estimate. Taken together, there might be little indication of publication bias in this meta-analysis.

Subgroup analyses for the association between physical activity and risk of lung cancer was shown in Table 2. Consistent with the overall analysis, a significant inverse association was observed in most of the subgroups. Compared with women with low level of physical activity, the pooled RRs for those with high level of physical activity were 0.90 (95%CI: 0.82, 0.99). The corresponding RR for men was 0.81 (95%CI: 0.73, 0.90). The RR for leisure time physical activity was 0.81 (95% CI: 0.71, 0.93), which was comparable with total physical activity (RR = 0.81, 95% CI: 0.71, 0.93). Smokers with a high level of activity was associated with a 10% lower in risk of lung cancer risk (RR = 0.90, 95% CI: 0.84, 0.97), while the association was not significant among non-smokers (RR = 0.95, 95% CI: 0.88, 1.03). Of note, subgroups with whether or not adjusted for smoking intensity and durations yielded the same magnitude of RR. However, the RR for subgroups without adjustment for dietary factors was 0.74 (95%CI: 0.71, 0.77), which was significantly lower than that with dietary factors adjusted (RR = 0.89, 95%CI: 0.84, 0.95).

**Table 2:** Subgroup results of association between physical activity and lung cancer risk.

**Subgroups**	**Number of studies**	**Pooled RR (95% CI)**	***I*^**2**^ (%)**	*P*-value^*^	***P*-value^^†^^**
Gender					0.265
Men	14	0.81 (0.73, 0.90)	47.4	0.025	
Women	8	0.90 (0.82, 0.99)	0	0.984	
Location					0.500
United States	8	0.86 (0.76, 0.96)	53.1	0.037	
Europe	8	0.81 (0.70, 0.94)	0	0.658	
Asia	2	0.91 (0.75, 1.10)	43.4	0.171	
Others	2	0.77 (0.70. 0.85)	62.9	0.101	
Size of Cohorts					0.473
<50,000	12	0.79 (0.68, 0.91)	41.1	0.06	
≥50,000	8	0.85 (0.78, 0.94)	73.1	< 0.001	
Type of physical activity					0.787
Total physical activity	11	0.81 (0.71, 0.93)	41.6	0.057	
Leisure time physical activity	9	0.81 (0.77, 0.93)	71.5	< 0.001	
Outcome measurement					0.726
Cancer registry	16	0.84 (0.77, 0.92)	66.5	< 0.001	
Others	4	0.81 (0.70, 0.97)	19.9	0.29	
Smoking status					0.598
Smokers	6	0.90 (0.84, 0.97)	36.5	0.164	
Non-smokers	5	0.95 (0.88, 1.03)	24.3	0.259	
**ADJUSTMENT FOR POTENTIAL CONFOUNDERS OR RISK FACTORS**					
Smoking intensity/durations					0.761
No	12	0.84 (0.77, 0.93)	69.9	< 0.001	
Yes	8	0.84 (0.74, 0.95)	17.1	0.291	
Body mass index					0.247
No	5	0.79 (0.71, 0.87)	39.1	0.145	
Yes	15	0.86 (0.80, 0.93)	34.4	0.082	
Alcohol drinking					0.432
No	10	0.82 (0.75, 0.91)	21	0.243	
Yes	10	0.86 (0.77, 0.96)	73.7	< 0.001	
Dietary factors					0.002
No	9	0.74 (0.71, 0.77)	0	0.571	
Yes	11	0.89 (0.84, 0.95)	25.1	0.197	

* P for heterogeneity within subgroups.

† *P for heterogeneity between subgroups*.

In sensitivity analyses, we recalculated the pooled RRs by sequentially excluding one study. The pooled RRs ranged from 0.82 (95% CI: 0.76, 0.89) to 0.86 (95% CI: 0.81, 0.92). The trend was generally similar with the overall analysis (Supplementary Figure 1).

## Conclusions

In summary, this meta-analysis suggest that the increased physical activity might be associated with lower risk of lung cancer. Such inverse association was identified among smokers rather than non-smokers. Large interventional studies are expected to further verify these findings. If the inverse association, as well as the effect modification by smoking status, reflects a causal relation, future precision prevention of lung cancer by increased physical activity may be more effective when targeting to smokers rather than non-smokers.

## Discussion

### Summary of Main Findings

In this meta-analysis of cohort studies, there was an inverse association between physical activity and risk of lung cancer. Increased physical activity may be associated with lower risk of lung cancer. The strength of association in men was stronger than that in women. Similar inverse association was observed in smokers, whereas no significant associations were found in never smokers. The observed inverse associations were robust across subgroups and sensitivity analyses.

Previous meta-analysis including both case-control studies and cohort studies suggested that regular recreational physical activity may be associated with reduced risk of lung cancer, with a pooled RR of 0.76 (95% CI: 0.69–0.85) (14). This pooled result was attenuated in current study with only cohort studies included in the meta-analysis. This inconsistence may be due to large numbers of case-controls studies included in previous meta-analysis, and the case-control design may be more likely to expose to high risk of biases, such as selection bias, recall bias and inverse causal bias, which may distort the true association. Furthermore, previous meta-analysis concluded that the risk was lower in women than that in men (RR: 0.68 for women, 0.85 for men) (15), which was inconsistent with the results of current study (RR: 0.90 for women: 0.81 for men) with the up-to-date evidence included in the analysis.

In current meta-analysis, there were significantly heterogeneous results across subgroups stratified by whether the included studies adjusted for the dietary factors. Although both of the subgroups showed significant results, the strength of the association differed. The dietary factors such as total energy intake may mutually interact with physical activity and be associated with lung cancer as either a confounder or an intermediate factor, which may subsequently lead to the heterogeneous results across subgroups. Moreover, heterogeneity may also exist across the subgroups stratified by smoking status. A significant inverse associations between physical activity and lung cancer risk were observed among smokers, but not among never smokers. One of the largest study (6) with pooled data of 12 prospective US and European cohorts observed a significant effect modification by smoking status, with an inverse association in smokers rather than non-smokers. Of note, previous studies explained the heterogeneity as a result of residual confounding by smoking intensity and durations. However, we stratified by whether the included studies adjusted for the smoking intensity and durations in current meta-analysis, yielding the similar RR of 0.84 in both subgroups, which may rule out the possibility of residual confounding by smoking intensity.

It is suggested that physical activity increases pulmonary function, which may reduce the duration of exposure to carcinogenic agents in the lung (41). Several epidemiologic studies have shown that elevated lung function is associated with reduced lung cancer risk (41–43). Randomized trials showed that a 1 year physical activity intervention reduced levels of estrone and estradiol, which play key roles in lung carcinogenesis and lung cancer growth (41, 44). In addition, multiple potential biological mechanisms, including inflammation, and oxidative stress have long been hypothesized underlying the observed association between physical activity and lung cancer (41, 42). Physical activity may decrease body fat, interleukin-6 and tumor necrosis factor-α (42). The decreased levels of interleukin-6 and tumor necrosis factor-α may be associated with lower risk of lung cancer (42). Previous study indicated that more frequent physical activity was associated with a lower odds of having an elevated C-reactive protein, fibrinogen and reactive oxygen species levels (45). The increased levels of reactive oxygen species are extensively involved in the mechanisms of chronic lung inflammation and thus contribute to the development of lung cancer (46, 47). However, these biological mechanisms oxidative stress and cancer development are still controversial. Oxidative stress can cause tumor initiation if they damaged DNA; however, oxidative stress has also been shown to help kill early tumor cells in process of tumor promotion (48). Since the exact mechanism of physical activity on lung carcinogenesis remains unclear, further research is still warranted.

### Limitations

Our study also has some limitations. First, because of the nature of the observational design, residual confounding may distort the observed association and we were not able to address problems with confounding inherent in the original studies. For example, dietary factors such as total energy intake may affect physical activity and be associated with risk of lung cancer. The lack of data on chronic lung disease may probably bias the results. However, most studies included in this meta-analysis adjusted for some of the major potential confounders, such as age, sex, smoking status, body mass index. To overcome this limitation, large randomized trials would be necessary to definitively verify the observed association. Second, there was a significant heterogeneity in current meta-analysis. There are several potential explanations for the observed between-study heterogeneity, such as different type of physical activity, different measurement of physical activity, various size of cohort and length of follow-up from study to study. Since all these together may result in statistical heterogeneity, the results of current study should be interpreted with caution. Despite these limitations, the major strength of this meta-analysis was the large sample size which provided sufficient statistical power to detect a significant association, as well as across various subgroups.

## Author Contributions

YaL and X-XF contributed to the conception or design of the work. X-XF and YaL contributed to the acquisition, analysis, or interpretation of data for the work. YaL drafted the manuscript. YuL and Y-PB critically revised the manuscript. All authors gave final approval and agree to be accountable for all aspects of work ensuring integrity and accuracy.

### Conflict of Interest Statement

The authors declare that the research was conducted in the absence of any commercial or financial relationships that could be construed as a potential conflict of interest.

## Abbreviations

RR: relative risk
CI: confidence interval.
