# Supplementary material for: Association Between Physical Activity and Lower Risk of Lung Cancer: A Meta-Analysis of Cohort Studies
Source: Front Oncol. 2019 Jan 22;9:5. doi: 10.3389/fonc.2019.00005 (PMC6349707; doi:10.3389/fonc.2019.00005)
Supplement: Supplementary file 1 [file Table_1.DOCX]

**Supplementary Table 1 Quality of the Cohort Studies Included in the Meta-analysis***

|  | **Selection** | | | | **Comparability** | **Outcome** | | |  |
| --- | --- | --- | --- | --- | --- | --- | --- | --- | --- |
| **Study** | **Representativeness of the exposed cohort** | **Selection of the non-exposed cohort** | **Ascertainment of exposure** | **Outcome of interest was not present at start of study** | **Comparability of cohorts on the basis of the design or analysis†** | **Assessment of outcome** | **Follow-up long enough for outcomes to occur‡** | **Adequacy of follow up of cohorts§** | **Total Score** |
| Albanes,1989 | 1 | 1 | 0 | 1 | 2 | 1 | 1 | 0 | 7 |
| Severson,1989 | 1 | 1 | 1 | 1 | 2 | 1 | 1 | 1 | 9 |
| Knekt,1996 | 1 | 1 | 1 | 1 | 0 | 1 | 1 | 0 | 6 |
| Thune, 1997 | 1 | 1 | 1 | 1 | 2 | 1 | 1 | 1 | 9 |
| Lee, 1999 | 0 | 1 | 1 | 1 | 2 | 0 | 1 | 1 | 7 |
| Wannamethee, 2001 | 1 | 1 | 1 | 0 | 2 | 1 | 1 | 1 | 8 |
| Colbert, 2002 | 1 | 1 | 1 | 1 | 2 | 1 | 1 | 0 | 8 |
| Alfano, 2004 | 0 | 1 | 1 | 0 | 2 | 1 | 0 | 1 | 6 |
| Schnohr, 2005 | 1 | 1 | 1 | 1 | 2 | 1 | 1 | 0 | 8 |
| Sprague, 2008 | 1 | 1 | 1 | 0 | 2 | 1 | 1 | 0 | 7 |
| Yun, 2008 | 1 | 1 | 1 | 1 | 2 | 1 | 1 | 0 | 8 |
| Inoue, 2008 | 1 | 1 | 1 | 1 | 2 | 1 | 1 | 1 | 9 |
| Laukkanen, 2010 | 1 | 1 | 1 | 1 | 2 | 1 | 1 | 1 | 9 |
| Land, 2014 | 0 | 1 | 0 | 1 | 2 | 1 | 1 | 1 | 7 |
| Sormunen,2014 | 0 | 1 | 1 | 0 | 1 | 1 | 1 | 0 | 5 |
| Moore, 2016 | 1 | 1 | 1 | 1 | 2 | 1 | 1 | 0 | 8 |
| Wang, 2016 | 0 | 1 | 1 | 1 | 2 | 1 | 1 | 0 | 7 |
| Patel, 2017 | 1 | 1 | 1 | 1 | 2 | 1 | 1 | 0 | 8 |
| Laaksonen, 2018 | 1 | 1 | 1 | 1 | 2 | 1 | 1 | 0 | 8 |
| Borch, 2018 | 1 | 1 | 1 | 1 | 2 | 1 | 1 | 0 | 8 |

* A study could be awarded a maximum of one point for each item except for the item “Comparability of cohorts on the basis of the design or analysis”

**†** A maximum of 2 points could be awarded for this item. Studies that included adjustment for age, sex (if applicable) and smoking status or intensity received one point, and studies that included most of the other important confounders such as body mass index, alcohol, dietary factors received an additional point.

‡ A cohort study with a follow-up time >5 years was assigned one point.

**§** A cohort study with a follow-up participation rate >85% was assigned one point.
