# Supplementary figures and images for: Association Between Physical Activity and Lower Risk of Lung Cancer: A Meta-Analysis of Cohort Studies
Source: Front Oncol. 2019 Jan 22;9:5. doi: 10.3389/fonc.2019.00005 (PMC6349707; doi:10.3389/fonc.2019.00005)

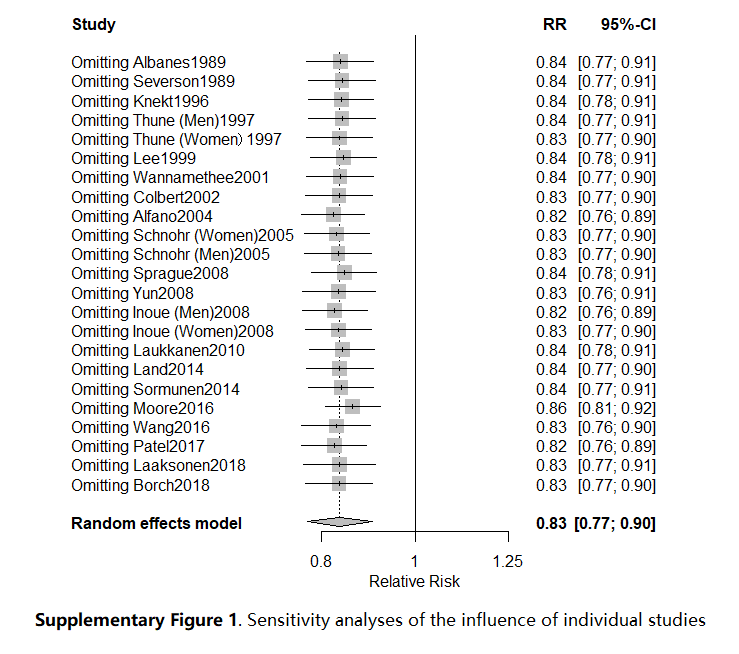

Supplement: Supplementary file 2 [file Image_1.tiff]
